# A new selective force driving metabolic gene clustering

**DOI:** 10.1128/msystems.00960-24

**Published:** 2024-10-28

**Authors:** Marco Fondi, Francesco Pini, Christopher Riccardi, Pietro Gemo, Matteo Brilli

**Affiliations:** 1 Department of Biology, University of Florence, Florence, Italy; 2 Department of Biosciences, Biotechnology and Environment (DBBA), University of Bari Aldo Moro, Bari, Italy; 3 Department of Biosciences, University of Milan, Milan, Italy; Rice University, Houston, Texas, USA

**Keywords:** operon accretion, gene clustering, metabolic control analysis, genomics, DNA replication

## Abstract

The evolution of operons has puzzled evolutionary biologists since their discovery, and many theories exist to explain their emergence, spreading, and evolutionary conservation. In this work, we suggest that DNA replication introduces a selective force for the clustering of functionally related genes on chromosomes, which we interpret as a preliminary and necessary step in operon formation. Our reasoning starts from the observation that DNA replication produces copy number variations of genomic regions, and we propose that such changes perturb metabolism. The formalization of this effect by exploiting concepts from metabolic control analysis suggests that the minimization of such perturbations during evolution could be achieved through the formation of gene clusters and operons. We support our theoretical derivations with simulations based on a realistic metabolic network, and we confirm that present-day genomes have a degree of compaction of functionally related genes, which is significantly correlated to the proposed perturbations introduced by replication. The formation of clusters of functionally related genes in microbial genomes has puzzled microbiologists since their first discovery. Here, we suggest that replication, and the copy number variations due to the replisome passage, might play a role in the process through a perturbation in metabolite homeostasis. We provide theoretical support to this hypothesis, and we found that both simulations and genomic analysis support our hypothesis.

**IMPORTANCE:**

The formation of clusters of functionally related genes in microbial genomes has puzzled microbiologists since their discovery. Here, we suggest that replication, and the copy number variations due to the replisome passage, might play a role in the process through a perturbation in metabolite homeostasis. We provide theoretical support to this hypothesis, and we found that both simulations and genomic analysis support our hypothesis.

## INTRODUCTION

Operons (1) are one of the hallmarks of prokaryotic genomes; in their most basic form, they comprise a single promoter at the 5′ end, followed by two or more genes and a transcription terminator at the 3′ end, such that they are transcribed into polycistronic messenger RNAs. Additional regulatory sites (alternative and/or internal promoters, attenuators, etc.) can be present to provide fine control over expression levels (2, 3). Genes in the same operon often participate in the same functional process.

Although common in prokaryotes, there are exceptions or peculiarities (4, 5) in eukaryotes; the smaller effective population sizes of many eukaryotes, according to the drift model, would lead to operon disruption; moreover, differences related to cell cycle and the transcription/translation processes would not probably make operons effective as they are in prokaryotes (6).

The evolution of operons has been debated since their discovery; early ideas often coupled operon formation and metabolic pathway evolution, but although pathways are often ancient, taxonomic variability in operon organization of the corresponding genes in different taxa suggests a more recent evolutionary history (7).

The process of operon evolution can be split into two aspects: one is assembly, by which scattered genes form clusters that may then evolve into operons. The second is maintenance in evolutionary time, which depends on the fitness differential provided by the operon with respect to the same genome with scattered genes. Since what makes an operon advantageous once it is formed is not necessarily what drove the genes at nearby loci, its assembly and maintenance may be consequences of very different forces. For instance, an operon may reduce noise in protein abundances as previously suggested (8), but this advantage can plausibly be selected for by evolution only after the genes get close enough on the genome and much less during the intermediate steps.

Models dealing with the identification of the force driving gene clustering during the formation of operons exist. The Fisher model suggests that the compactness of operons may reduce the chances of destructive recombination events (9), an idea recently revived in (10) where the authors used simulations to propose that random gene deletion could drive the formation of functionally related gene clusters. The co-regulation model is intrinsically linked with the operon rationale: genes stay together to facilitate their coordinated expression (11); however, others have shown that the formation of operons for the purpose of co-regulation is both unnecessary and implausible (12, 13) because independent promoters can in principle evolve characteristics enabling the co-expression of genes encoded at different loci. Moreover, although co-regulation is very likely one of the main reasons for maintaining operons once they exist, it provides no clue about how genes would get close enough to form a single transcriptional unit. The selfish operon model (12) focuses on the formation of operons by deletion of intervening genes after horizontal gene transfer of a much larger fragment. However, this hypothesis was criticized on the basis of a high degree of gene clustering for housekeeping genes, whose horizontal transfer is infrequent (14). Another possible explanation is that the coupling of transcription and translation, together with a strong limitation of diffusion by molecular crowding, would confine the enzymes in a relatively small volume of the cytoplasm, maximizing interactions and metabolic fluxes (15–17). A similar idea forms the basis for the protein immobility model (PIM) (18) where gene clustering is beneficial because it reduces metabolic costs. In this model, the source and sink of a two-enzyme metabolic pathway are at fixed positions in the cell. Under this view, a gradient of the intermediate builds up in between, and therefore, the amount of intermediate required for such a gradient is much smaller if the enzymes are close. However, not all proteins encoded by the same operon interact, channeling of metabolic intermediates is not so widespread, and recent estimates, withstanding the reduced mobility of proteins in the cell with respect to pure water, suggest that two particles can find each other in the cell in a matter of seconds (19), a much shorter time scale than the characteristic time of metabolic systems. By using stochastic simulations of simple biochemical systems, evidence was found for noise reduction in the abundance of proteins encoded by operons; however, this was limited to some type of interaction established by the products (8). It should be added that the two hypotheses mentioned above strongly rely on a strict coupling of transcription and translation that might not be as general as previously assumed (20). Genome size and/or its proxies were also suggested to be markers of the intensity of natural selection for operon organization, under the idea that since larger genomes have more complex genetic networks thanks to the presence of more transcription factors, they would endure weaker selection for operons than smaller genomes, where regulation alternatives are scarce (21).

Several hypotheses have been proposed to explain the molecular mechanisms responsible for clustering genes. Duplication and divergence are at the basis of the Natal model, with metabolic pathways evolving in a step-wise fashion; the same idea is also central in the Piece-wise model where operons are assembled by clustering of sub-operons originated by in-tandem duplications of ancestral genes (7, 22). In the SNAP hypothesis, gene order rearrangements could be obtained by duplication events followed by selection during niche adaptation where non-selected genes are lost or inactivated (23). Operon formation could also take place on plasmids which may work as scribbling pads (24). A recent proposal is based on consecutive reactions of insertion, deletion, and excision of insertion sequences, the IDE model (25).

Works on this topic generally focus on the force driving gene clustering or on the mechanisms by which genes can get clustered. The former assumes that molecular mechanisms for rearranging gene order exist and focuses on plausible fitness functions, whereas the latter often assume some fitness functions and focus on the ability of different types of genome rearrangements to build gene clusters (26, 27). The lack of a single theoretical framework exhaustively accounting for the hows and whys of gene cluster assembly and maintenance suggests that different forces might be involved in this process, and each, under appropriate conditions, may lead to gene clustering and operon formation, as also concluded by a real study-case (28). Here, we propose a novel selective force for the assembly of metabolic gene clusters and operons, which is naturally active with varying intensity in all prokaryotic species. More specifically, we suggest that metabolic gene clusters are evolutionary solutions that minimize the perturbations on metabolite homeostasis introduced by DNA replication.

**Fig 1:**
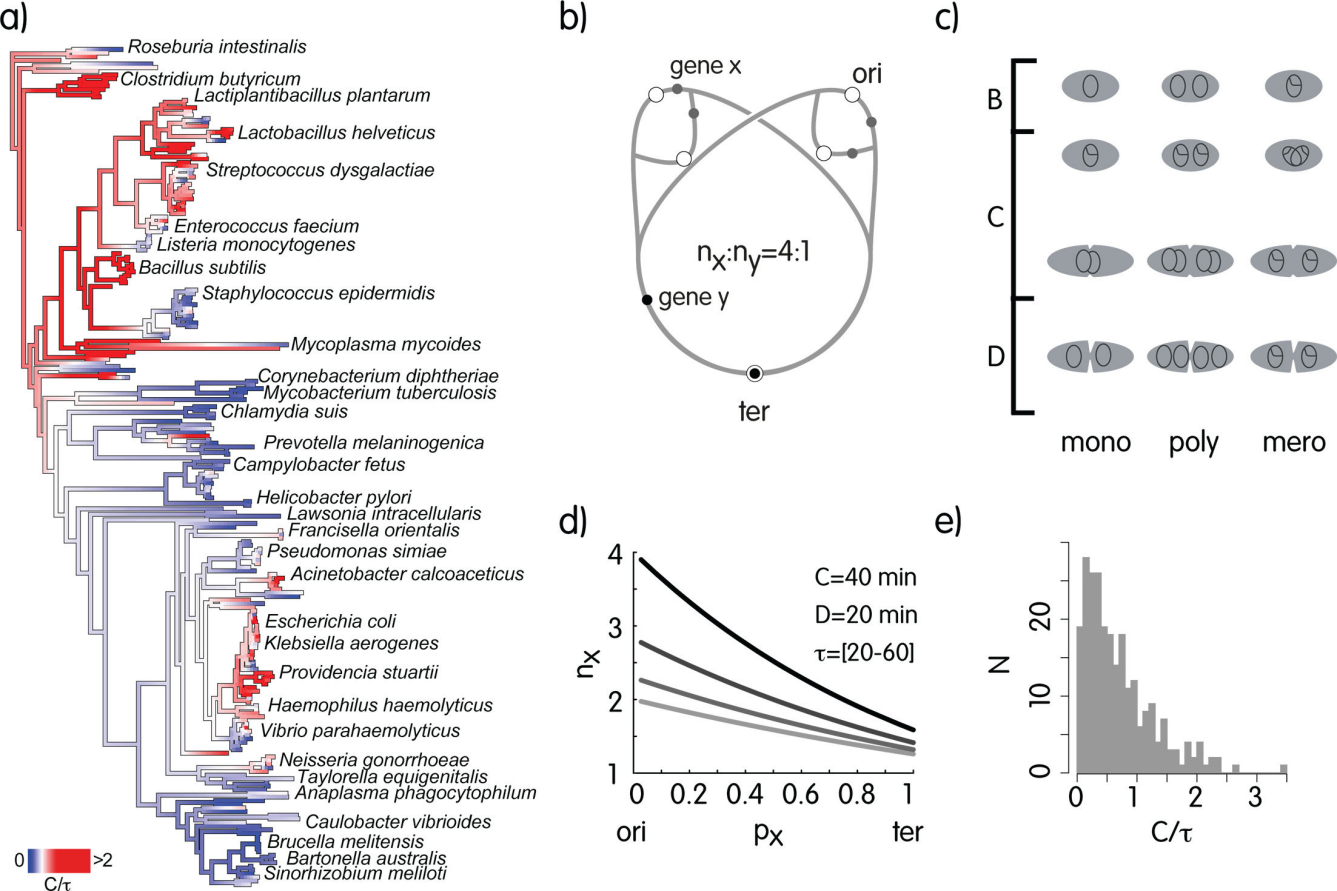
(a) Mapping ${log}_{2}\;n_{ori}/n_{ter}={log}_{2}PTR=C/\tau$ on a phylogenetic tree of Bacteria. Only a few species are indicated for clarity and ${log}_{2}\;n_{ori}/n_{ter}$ is truncated at 2 to highlight differences, whereas $PTR_{max}\approx 10$. Mono- and poly-ploids have PTR ≤ 2 (equal sign when all chromosomes in all cells of the population are under replication); mero-oligoploids can have PTR > 2, but not so at low growth rates and are therefore able to switch from one growth mode to another. (b) Replication and its effect on copy number of loci with three replisomes as in the scheme, *x* and *y*, are in a 4:1 ratio, but this changes together with the number of replisomes. (c) Different modes of growth in prokaryotes. (d) Average copy number of loci as a function of the position on the Ori-Ter axis and division time (dark, shorter division time). (e) Distribution of the average C/τ ratio estimates for the species in panel a.

Bacterial species have a more or less strict coupling of DNA replication with cell division. Species like *Caulobacter crescentus* and *Staphylococcus aureus* lie at one extreme because they implement genetic programs that determine cell division just after the chromosome has been copied (29, 30) and are consequently strictly monoploid. These species can contain one or two chromosomes at most and one active replisome. Other species, like, for instance, the two model organisms *Escherichia coli* and *Bacillus subtilis*, are able to switch from monoploidy at slow growth rates to mero-oligoploidy during rapid growth. In mero-oligoploids, several replisomes can run concomitantly on the chromosome, enabling the bacterium to sustain division times shorter than the time for replicating the chromosome (31, 32). *E. coli* can for instance have up to 10 replication forks (Fig. 1a and b) (33). The number of active replisomes can therefore significantly change in many species, but although an increased number of replication forks has been related to shorter division times in certain organisms (34), the relationship seems to fail significance when distant taxonomic groups are analyzed together (35). Other bacterial species can be oligo- or poly-ploids, owning from a few to tens or hundreds of full chromosomes, like many cyanobacteria (36). In this case, depending on the species, replication can involve only one chromosome at a time, or many, and all of them, are usually expressed (37). Endosymbionts or giant bacteria show the most extreme situations as they can harbor thousands of chromosome copies (38, 39).

Replication activity can be investigated by using genome sequencing data and calculating the ratio of coverage at the Ori and Ter regions ($n_{ori}/n_{ter}$ ratio) (Fig. 1). The number of active replisomes in the artificial conditions where bacteria are grown prior to genome sequencing is likely rarely attained in the environment, and however, it is informative about the potential ability to implement a mero-oligoploid mode of growth by the different species. This ratio, often called the peak-to-trough (PTR) ratio in metagenomics, has recently been used to show that the growth rate of bacteria in the environment can change significantly (35, 40), which was confirmed by approaches not based on this ratio (41, 42).

Recently, the use of single-cell transcriptomic data demonstrated that replication imparts a clear pattern to gene expression, at least in *E. coli* and *S. aureus*. Moreover, the same authors showed that this pattern is also detectable in *C. crescentus* gene expression data of a synchronized population (43). We therefore conclude that (i) when the growth rate changes, loci experience variations in copy number depending on their distance from the origin, and (ii) this affects the abundance of transcripts (44, 45), with maybe a partial buffering by global regulatory mechanisms, like super-coiling (46).

Here, we provide theoretical considerations about the effect of active DNA replication on metabolic homeostasis. Under this view, replication can provide a selective force for driving functionally related genes at nearby loci in evolution, which we interpret as a fundamental pre-requisite for operon formation.

## MATERIALS AND METHODS

### PTR calculation, models, and optimizations

Identification of origin and terminus for each genome was done with function oriloc from package seqinr (47). Coverage around the two loci was obtained by first extracting a 50-kb region centered on the Ori (Ter) locus and then mapping publicly available genome sequencing data from SRA using Salmon (48) in mapping mode. Only species with at least five sequencing libraries available were considered. Coverage was not normalized because we focused on the ratio at the two loci. The R function t.test was used for evaluating log_2_PTR larger than 0 (PTR > 1) or larger than 1 (PTR > 2), the latter identifying mero-oligoploid species, and it was also used to get 95% confidence intervals of the mean values. The two gene-system was implemented in Matlab. The scripts implementing the models are available on Mendeley Data, doi: 10.17632/67txzm96jg.1.

### Linlog metabolic model

The linlog format for metabolic systems is a linear alternative to model enzymatic rates (49); it was developed to overcome the problems deriving from the non-linearity of expressions like Michaelis-Menten rate equations such as the difficulty in parameter identification and the impossibility of obtaining analytical solutions even for systems of only a few equations. In the linlog, each rate is modeled as a linear combination of logarithms of metabolite concentrations; therefore, we can obtain an analytical solution for the steady state (equation 5). Using this approximation, rates can be modeled as:


(1) $$v=E\left(A^{\prime }+B^{\prime }logx+C^{\prime }logc\right).$$

where ${𝑬}_{r\times r}$ is a diagonal matrix of enzyme abundances, ${{𝑨}^{\prime }}_{r\times 1}$, ${{𝑩}^{\prime }}_{r\times m}$, and ${{𝑪}^{\prime }}_{r\times 2}$ contain parameters, ${𝒙}_{m\times 1}$ is the vector of variable metabolite concentrations, and ${𝒄}_{2\times 1}$ the vector of metabolites that are external to the system (in our case glucose and acetate are parameters). In the original formulation, both rates and concentrations are normalized by their values in the reference condition (indicated by apex 0, below), but here, we integrate the reference level with the parameters to work with absolute quantities. More in detail:


(2) $$A^{\prime }=(J^{0}/E^{0})\;(1-Blogx^{0}-Clogc^{0})$$

(3) $$B^{\prime }=(J^{0}/E^{0})$$

(4) $$C^{\prime }=(J^{0}/E^{0})\;C,$$

where $1_{r\times 1}$ is a vector of ones, ${{𝑱}^{0}}_{r\times 1}$, ${{𝒙}^{0}}_{m\times 1}$, and ${{𝒄}^{0}}_{2\times 1}$ are the vectors of steady state fluxes, variables, and external metabolite concentrations for the reference condition, respectively. ${𝑩}_{r\times m}$ and ${𝑪}_{r\times 2}$ are the matrices containing elasticities with respect to variable and external metabolites, respectively (49). Given the stoichiometry matrix ${𝑵}_{m\times r}$ of the system, which associates enzymes to specific biochemical reactions where metabolites are consumed and produced, we can solve the system for the steady state (when $\frac{d𝒙}{dt}=𝑵𝒗=0$):


(5) $$logx=-{\left(NEB^{\prime }\right)}^{-1}(NEA^{\prime }+NEC^{\prime }logc).$$

Using equation 5, one can input enzyme levels to obtain steady state concentrations of the metabolites. The model is not parameterized with respect to experimental data, but it has biologically meaningful parameters, and therefore, we use it as a benchmark for testing our hypothesis in a realistic model and not to derive data for comparison with true genomes. We note that although the elasticities defined in metabolic control analysis (MCA) depend on the concentration of metabolites, here, they are fixed parameters. We are therefore assuming that the dependencies of an enzyme on substrate(s) and product(s) do not change during growth at different rates, which is a limitation of the formalism used. Nevertheless, the linlog has a relatively wide range of validity around the reference point where it is calculated (50). The use of a metabolic network in this context is instrumental to model a realistic response to perturbations, for the presence of systemic properties that were described by metabolic control analysis (51).

### Optimization of gene positions

The optimization-based simulation is carried out using the R function nlminb and the average coefficient of variation in metabolite concentrations when division times are changed as the objective function (see text). We consider the inverse of this value as a proxy for fitness; therefore, genomes with smaller variability in metabolite concentrations have larger fitness. Genes can move on the genome under the hypothesis that genome organization should minimize perturbations to homeostasis, but no mechanistic model for genome organization evolution is explicitly implemented. The same approach is also used to test the optimization of promoters. In this case, genes cannot move, but a multiplier of each rate function simulates the strength of the promoter and is the parameter undergoing optimization. We highlight that each promoter has its own k to be optimized. In both cases, we use the following options: abs.tol = 1E−15, xf.tol = 1E−12, iter.max = 1E+06, eval.max = 1E+06, x.tol = 1E−12, step.min = 1E−06, and step.max = 1.

### Simulation of gene order evolution

This simulation implements a more mechanistic model of structural rearrangements, with both translocations and inversions. Overall, in this simulation, genes coding for the enzymes of the metabolic model are randomly placed on a virtual ori/ter axis. Their position defines the multiplicity of loci across the growth rates that we test and can be translated into enzyme abundances, thanks to the Helmstetter and Cooper model (31) and assuming proportionality of copy numbers and gene expression level. At each growth rate, enzyme abundances are fed to the steady state solution of the metabolic model, and metabolite concentrations for the new steady state are retrieved. Once this procedure is done for all growth rates under test, we calculate the coefficient of variation for all metabolites across the growth rates and average them, and the optimization tool then works to minimize it by rearranging the gene order at each iteration. This general concept was implemented as follows. A genome is defined as a circular graph with 600 loci, comprising the origin, the terminus, and the 34 target genes coding for the enzymes of the metabolic model. The origin is defined a priori, whereas the terminus is assigned to a node approximately equidistant from the origin. All genes are separated by 10k nucleotides to adjust the size of the artificial genomes to the Helmstetter and Cooper model (31), according to which the time for a replication point to traverse the genome and the time between the end of a round of replication and cell division can describe the replication of the bacterial genome during the division cycle. This introduces a limit in the simulation, as genes cannot get closer than this distance. The fitness of genomes is the same as in the previous simulations, that is proportional to the inverse of the average coefficient of variations of metabolite concentrations when the division time changes. The tested division times are [20, 30, 40, 50, 70, 90, 120] minutes. This range encompasses biologically plausible values for a bacterium like *E. coli* and many others, switching from fast (τ = 20 min or μ = 2.07 h^−1^) to relatively slow growth (τ = 120 min or μ = 0.345 h^−1^). The simulation starts with 10^5^ identical genomes that, at each generation, can undergo structural rearrangements with certain probabilities. In the simulations presented here, we tested two different scenarios for inversions and translocations, $p_{inv}=[0.005,0.0025]$ and $p_{transl}=p_{inv}/2$. Once an inversion occurs, it moves blocks of max(1,N(mean=5,var=9)) genes, whereas translocations move blocks of max(1,N(mean=3,var=9)) genes. After all genomes undergoing an event are processed, fitness values are re-calculated on the basis of each genome structure, and all variants below the 50th percentile are removed. During the simulation, genomes where the minimum Ori-Ter distance is less than 75% of the original distance are assigned a very low fitness and are consequently removed from the population to ensure that the structure of the chromosome is conserved.

The remaining genomes undergo (i) grouping into unique structural variants and (ii) sampling, proportionally to their fitness to re-generate the population of 10^5^ individuals for the next generation. During the simulation, we keep trace of the number of pure clusters defined as contiguous stretches of at least two genes from the metabolic network and the size of the largest cluster. We also track mixed clusters that have target metabolic genes at the extremities and at most two consecutive non-target genes inside.

### Gene clustering analysis and proximity score calculation

The Kyoto Encyclopedia of Genes and Genomes (KEGG) (52) provides a collection of pathway maps representing our knowledge of the molecular interaction, reaction, and relation networks for a number or biologically relevant areas of research, such as metabolism or cellular processes. Each KEGG pathway also contains manually defined functionally tight gene sets of different types and scopes. Metabolic modules are assigned to the pathway module category, on which we focus our attention in this work. For every species under analysis (181), we obtained all gene-to-gene distances within each pathway module and then calculated the first quartile after processing all modules. This number represents the gene-to-gene distance such that 25% of all distances are smaller. The logarithm base 2 of the reciprocal of this number is the proximity score (PS) of a species that we contrast to the PTR. Scripts implementing these analyses and the associated data are available on Mendeley Data, doi: 10.17632/67txzm96jg.1.

## RESULTS

### Metabolic consequences of chromosome replication

In this section, we provide the theoretical foundations of our hypothesis by linking together chromosome replication and its possible effects on cellular homeostasis. We do this by integrating, for the first time, the so-called PTR (35) with MCA, an established theoretical framework focused on understanding the control of metabolic fluxes to predict the response to perturbations. In short, MCA is a sensitivity analysis providing interesting insights into the response of metabolic systems to infinitesimal or macroscopic perturbations of enzyme levels. Experimental evidence supporting the main conclusions of MCA has accumulated since its first description (for instance, references 53, 54). The PTR can be calculated using genome sequencing data and knowledge about the position of the origin and the terminus in the genome. Fig. 1a shows the PTR across a phylogenetic tree of bacteria, highlighting that both firmicutes and enterobacteria are mostly mero-oligoploid, whereas for instance, alphaproteobacteria are known to be mono-ploid or poly-ploid and have low PTR values. We noted that certain species, like *S. aureus* likely evolved mono-ploidy from a mero-oligoploid ancestor. If the PTR was constant (Fig. 1b), the copy number of two genes *x* and *y* located at distant loci on the genome would also be constant. However, since the number of replisomes per chromosome can change in time, these genes experience not only different multiplicities in time but also varying relative abundances. For instance, in Fig. 1b with three replication forks, *x* and *y* are in a 4:1 ratio, but with one replication fork, the ratio becomes 2:1. Since the multiplicity of genes affects the abundance of transcripts (43), the relative expression level of enzymes, especially when they are encoded by distant loci, is consequently affected. The formalization of the metabolic consequences of large changes in the abundance of enzymes belonging to the same metabolic pathway is one of the fundamental results of MCA (equation 6) (55). For metabolic flux, the so-called flux amplification factor is


(6) $$\frac{J^{r_{i}}}{J^{0}}=\frac{1}{1-\sum_{i=j}^{m}C_{E_{i}}^{J^{0}}\frac{r_{i}-1}{r_{i}}}\;\;,$$

where the Js are steady state fluxes for the reference (${}^{0}$) or the new steady state, induced by changing the abundance of m−j enzymes in a pathway by different relative factors $r_{i}=\frac{[E_{i}{]}^{new}}{[E_{i}{]}^{old}}$, and $C_{E_{i}}^{J^{0}}$ is the flux control coefficient (FCC) (51) of enzyme $E_{i}$ on the pathway, a key quantity for understanding metabolic control, which is defined in equation 7.


(7) $$C_{E_{i}}^{J}=\frac{\partial J}{\partial E_{i}}\frac{E_{i}}{J}=\frac{\partial lnJ}{\partial lnE_{i}}.$$

In practice, the FCC of enzyme i tells us about the fractional change in pathway flux elicited by a fractional change in $E_{i}$. Importantly, FCCs are systemic quantities that can be measured only in the full metabolic system. Nevertheless, the so-called summation theorem (51):


(8) $$\sum_{i=1}^{n}C_{E_{i}}^{J}=1,$$

with n representing the number of enzymes in the pathway; this constrains the values of the FCCs, since they have to sum up to 1. This theorem, at the same time, demonstrates that fluxes are not usually modulated by key rate limiting enzymes; instead, control is shared by several (potentially all) enzymes in the metabolic network (51). For this reason, FCCs can be calculated only in the intact (*in vivo*) system. A consequence of equation 8, confirmed by many *in vivo* measurements since early times, is that the flux control coefficient of an enzyme with respect to a pathway is small on average (55–57). Therefore, when the abundance of one enzyme in a pathway is changed in isolation (i.e., the other enzymes don’t change), the flux will be hardly affected, whereas when all enzymes of a pathway are changed by the same factor $r_{i}=r$ (coordinate change), the system relaxes to a new steady state where the flux is scaled by r but metabolite concentrations stay constant (perfect homeostasis). When the change in abundance of different enzymes is unequal, the ensuing change in flux will depend both on the FCCs and new enzyme levels. In this case, since enzyme rates are not scaled proportionally, metabolite concentrations will move to a new steady state, potentially breaking homeostasis. This effect on metabolites can also be quantified by a relationship focusing on metabolite concentrations when the abundance of one enzyme in a pathway is scaled by a relative factor r (57):


(9) $$\frac{S^{r}}{S^{0}}=\frac{1-(C_{E}^{J}-C_{E}^{S})\frac{r-1}{r}}{1-\frac{r-1}{r}C_{E}^{J}}.$$

$C_{E}^{S}$ is the concentration control coefficient (CCC) of the enzyme over metabolite S, defined similarly to the FCCs (51) but in relation to metabolite concentrations. In this case the Summation Theorem (58) states that


(10) $$\sum_{i=1}^{n}C_{E_{i}}^{S}=0.$$

Contrary to the summation theorem for fluxes, in this case there is no limitation to the magnitude of CCCs. When we change the abundance of an enzyme whose $C_{E_{i}}^{J}\approx 0$ (i.e., the enzyme has negligible control on the pathway’s flux) equation 9 becomes (59)


(11) $$\frac{S^{r}}{S^{0}}=1+\frac{r-1}{r}\;C_{E}^{S}.$$

Therefore, the manipulation of the abundance of an enzyme with negligible control over the flux of a pathway can nonetheless perturb metabolite pools by an arbitrarily large factor (59). Even with the limitations imposed by the system (through equation 8 and equation 10), cells manage to modulate fluxes as a function of the demand while keeping metabolite concentrations within acceptable limits.

This can theoretically be achieved by making so that abundance changes of functionally related genes are coordinated, for instance, by putting them under the control of a same regulator—as for yeast’s amino acid biosynthetic genes, which are controlled by GCN4 (60). We are here concerned with the effect on metabolic state caused by variations in enzyme abundance that are driven by the copy number changes introduced by replication. To explore this issue, we integrate the effect of replication into equation 6 by exploiting classical models that connect the division time and the number of active replication forks (31, 61, 62). Indeed, when the duplication time (τ below, e.g., in minutes), the time required to replicate DNA and the delay of cell division after the genome has been replicated (C and D in e.g. minutes) are known, the average number of copies of locus x in the population can be predicted by


(12) $$n_{x}=2^{[(1-p_{x})C+D]/\tau }$$

where $p_{x}$ is the fractional position of the gene such that $p_{ori}=0$ and $p_{ter}=1$ (62) (Fig. 1c). It is therefore easy to demonstrate that


(13) $${log}_{2}\frac{n_{ori}}{n_{ter}}=\frac{C}{\tau },$$

which enables to use a quantity that can be calculated from genome sequencing data for inferring division times *in vivo*. We note that only mero-oligoploid species can have $n_{ori}/n_{ter}>2$ or C/τ>1, that is, a DNA replication phase (C) longer than the division time.

A simple model for the abundance of a certain transcript x under the regulation of transcription factor y could be integrated with multiplicity of the x locus as follows:


(14) $$\frac{dx}{dt}=n_{x}f(y,p)-\gamma x,$$

where 𝒑 indicates the parameters of a regulatory function, often having a type II or III functional response (e.g., Hill functions with n=1 and n≥2, respectively), and γ is the degradation rate of the transcript, which is inversely proportional to the half-life of the transcript ($\gamma =ln2/t_{1/2}$).

At the steady state, $\frac{d𝒙}{dt}=0$, therefore, the abundance of x is


(15) $$x_{i}^{ss}=n_{i}\frac{f(y,p)}{\gamma }=2^{[(1-p_{i})C+D]/\tau }\frac{f(y,p)}{\gamma_{i}}$$

which clearly shows that the steady state level of a transcript is also determined by changes in the division time. We can therefore exploit equation 6 to check what happens when a bacterium changes division time from $\tau^{0}$ to $\tau^{k}$. In this case, the relative change in abundance of enzyme i is


$$r_{i}=\frac{x_{i}^{k}}{x_{i}^{0}}=\frac{\overset{n_{i}^{k}}{\overbrace{2^{[(1-p_{i})C+D]/\tau^{k}}}}}{2^{[(1-p_{i})C+D]/\tau^{0}}}\frac{f_{i}(y^{k},p)}{f_{i}(y^{0},p)}\frac{\gamma^{0}}{\gamma^{k}}=2^{\frac{\tau^{k}-\tau^{0}}{\tau^{k}\tau^{0}}[(1-p_{i})C+D]}\frac{f_{i}(y^{k},p)}{f_{i}(y^{0},p)}\frac{\gamma^{0}}{\gamma^{k}}.$$

This relative change due to replication can be introduced in equation 6 to predict the change in pathway flux when growth rate changes:


(16) $$\frac{J^{k}}{J^{0}}=\frac{1}{1-\sum_{i=j}^{m}C_{E_{i}}^{J^{0}}\left(1-\frac{n_{i}^{0}f_{i}(y^{0},p)\gamma_{i}^{k}}{n_{i}^{k}f_{i}(y^{k},p)\gamma_{i}^{0}}\right)}\;\;,$$

As expected, the amplification factor depends on the variation in copy numbers of the loci as a function of the division time, the activity and abundance of the regulator, and the degradation rates. To isolate the effect of replication from the rest, we will introduce several simplifications: (i) all enzymes of the pathway under analysis undergo a change in abundance ($\sum_{i=1}^{m}C_{E_{i}}^{J^{0}}=1$). Indeed, replication affects all genomic positions; (ii) genes encoding enzymes in the pathway have identical promoters and degradation rates, and (iii) the change in division time does not affect the abundance of regulator y and the degradation rates [$f_{i}(y^{0},𝒑)=f_{i}(y^{k},𝒑)=f(y,𝒑)$ and $\gamma_{i}^{0}=\gamma_{i}^{k}=\gamma$].

Under these assumptions, equation 16 becomes the following:


(17) $$\frac{J^{\tau }}{J^{0}}=\frac{1}{\sum_{i=j}^{m}C_{E_{i}}^{J^{0}}\frac{n_{i}^{0}}{n_{i}^{k}}}=\frac{1}{\sum_{i=j}^{m}C_{E_{i}}^{J^{0}}\;\;2^{\frac{\tau^{k}-\tau^{0}}{\tau^{k}\tau^{0}}[(1-p_{i})C+D]}},$$

which highlights that when a bacterium changes division time across conditions, then the ensuing variation in copy number can perturb metabolic fluxes. As our simplifications may appear unreal, we note that even without such strong assumptions (equation 16), replication still contributes to the input/output response of classical transcriptional regulation. It also backs up criticisms to the idea that the optimization of promoter sequences of scattered genes is as good of an option for bacteria as well as eukaryotes: even with identical promoters, variations in growth rate can change the relative abundance of loci, hampering the evolutionary optimization of promoters. Genome rearrangements are an additional factor contributing to this difficulty, since by shuffling genes, they create novel configurations that may drastically decrease the optimality of promoters.

Since (i) equation 11 shows that even enzymes with negligible flux control coefficient can perturb metabolite pools by arbitrarily large factors and that (ii) significant variations in metabolite concentrations in the cell can break down cellular homeostasis, we suggest that a possible solution worked out by evolution could be the formation of gene clusters and operons. In such a case, the positions ($p_{i}$) of all genes with control over a certain pathway flux (such that equation 8 holds) are practically the same, and equation 17 becomes the following:


(18) $$\frac{J^{\tau }}{J^{0}}=2^{\frac{\tau^{0}-\tau^{k}}{\tau^{k}\tau^{0}}[(1-p)C+D]},$$

which is exactly the factor used to change all the enzymes (equation changes, once we introduce the above simplifications). Therefore, if the positions of the genes belonging to the pathway are similar, when the division time changes, the pathway’s flux is scaled by a certain quantity without changes in metabolite concentrations.

### Gene proximity minimizes variations in metabolite homeostasis

Let us now introduce a toy pathway to discuss more thoroughly the points raised above:


(19) $$X_{in}\overset{{}^{k_{1}}}{\to }s\overset{{}^{k_{2}}}{\to }X_{out},$$

where $X_{in}$ and $X_{out}$ are external metabolites, and $k_{1}$ and $k_{2}$ are the rates of two enzymes (with concentrations $E_{1}$ and $E_{2}$) coded for by genes located at distances $p_{1}$ and $p_{2}$ from the origin. Using mass action, we can write this simple system as follows:


(20) $$\frac{ds}{dt}=E_{1}k_{1}X_{in}-E_{2}k_{2}s,$$

which can be solved analytically at the steady state (when ds/dt=0):


(21) $$s^{ss}=\frac{E_{1}}{E_{2}}\frac{k_{1}}{k_{2}}X_{in}.$$

Enzyme abundances ($E_{i}$s) can be replaced by the relation introduced in equation 12, and by partial derivation, we can calculate the scaled sensitivity of the concentration of metabolite s with respect to changes in growth rate:


(22) $$\frac{\partial \ln s^{ss}}{\partial \ln C/\tau }=\frac{C}{\tau }\;\ln2\;(p_{2}-p_{1})=\mu \;C\Delta p,$$

since by definition the growth rate is $\mu =\frac{\ln\;2}{\tau }$.

The scaled sensitivity of metabolite concentrations with respect to the division time therefore depends on the difference in relative position of the two genes (Δp) and the change in C/τ. Equivalently, the sensitivity depends on the relative delay in copying one gene with respect to the other $\left(\Delta t=\frac{Cp_{2}}{\tau }-\frac{Cp_{1}}{\tau }\right)$. If $p_{1}>p_{2}$, an increase in C/τ, or more intuitively a decrease in division time causes a depletion of s at steady state, whereas the opposite happens if the division time increases. However, if $p_{1}\approx p_{2}$, the sensitivity tends to zero, making the pool independent from changes in division time.

**Fig 2:**
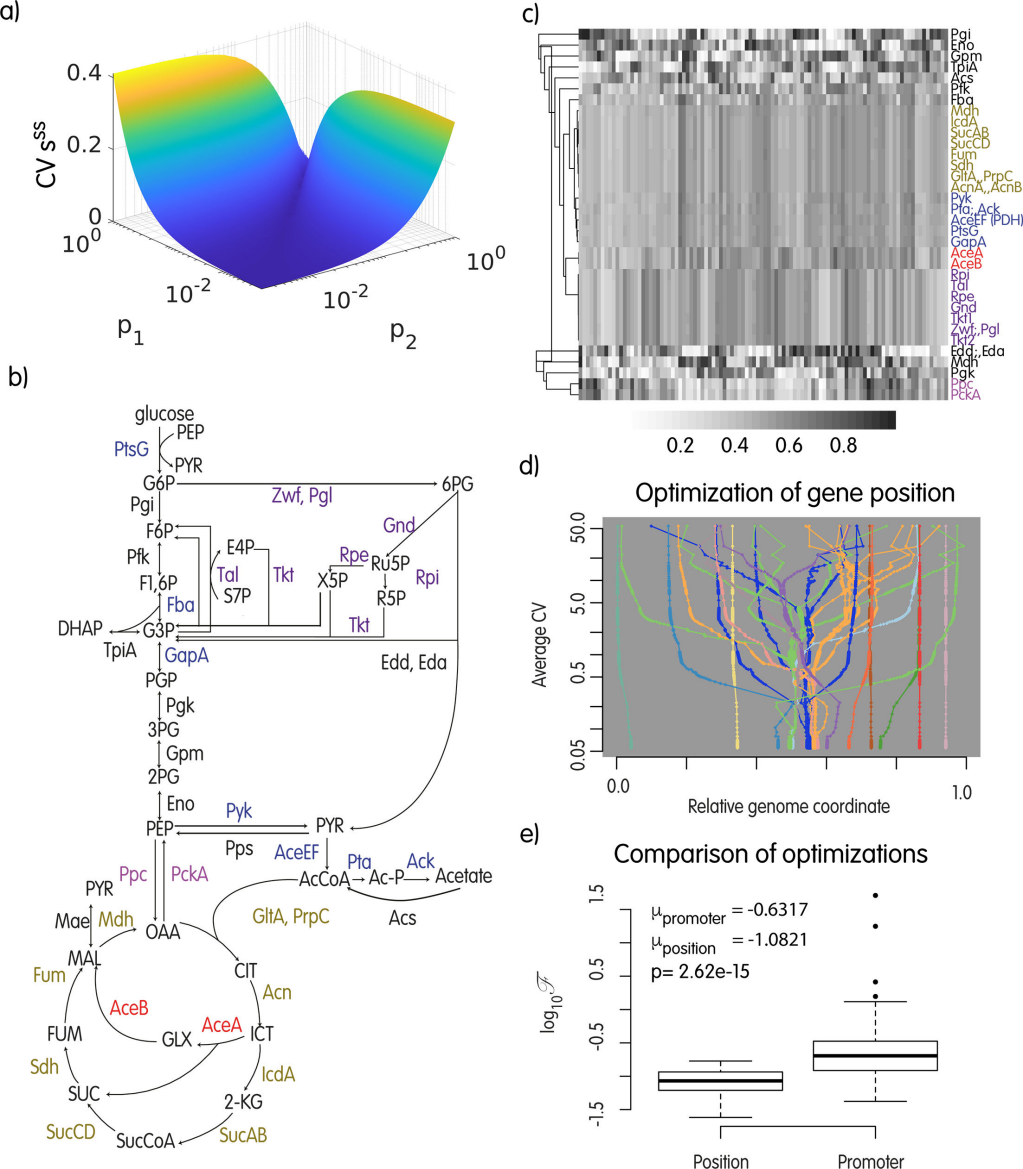
(a) Scanning of the genomic positions for the two genes in the toy model ($p_{1}$ and $p_{2}$, expressed as a distance from the terminus). $CVs^{ss}$ is the coefficient of variation of the concentration of metabolite s at steady state for 5 values of τ∈[20,60]. When the two genes are close (along the diagonal in the plot), perturbations to the homeostasis of s consequent to the changes in multiplicity of the genes τ are efficiently buffered. (b) The carbon utilization metabolic network implemented in the linlog model. (c) The results of 100 independent optimizations as described in the text and started from random gene positions. Rows are the enzymes in the model; the colormap indicates the position of the corresponding gene at convergence that is, same color in a column means the two genes are close on the chromosome. Groups of genes form similar clusters in different runs. (d) All iterations of one optimization run from panel c: genes ending in the same genomic region have the same color. Sub-clusters can form and destroy during the optimization, but once the final configuration is found, it is stable in time. (e) Comparison of the objective function at convergence for the optimization of gene positions and transcription rate constants (averages over 100 runs).

This basic example provides additional theoretical basis for a role on metabolite homeostasis by changes in division time. It is therefore plausible that events leading to the minimization of those perturbations have a positive impact on the fitness of a cell, thereby increasing the probability of fixation in the population. One of those mechanisms, as the above considerations suggest, is to group functionally related genes in a compact locus. To underline why, we refer to our toy model, and by scanning many positions for our two genes in a virtual 2 Mbp linear chromosome, we calculate a measure of the resulting variation in the abundance of metabolite s across a range of division times. Fig. 2a therefore confirms our theoretical prediction that when the division time changes, metabolite homeostasis is more precisely maintained if the genes are close on the genome; it also shows that the magnitude of perturbation is proportional to the relative distance of the genes, suggesting a mechanism for evolution to select for intermediate steps in the assembly of operons and gene clusters in general.

### Evolving operons *in silico*

The study of a simple two-genes system provided support to the idea that operons might be instrumental for metabolic homeostasis when the bacterium is actively replicating the genome, and more importantly, it changes growth rate. However, MCA has demonstrated that the metabolic response to perturbations in enzyme abundances has both local and global components (51). The former depends on enzyme characteristics, such as the catalytic mechanism with the associated genetically defined parameters (for instance $K_{m}$ and $V_{max}$), whereas the latter are determined by constrains that emerge as a consequence of the topology of the metabolic network, as elegantly shown by (63). Therefore, to understand the effect of perturbations, one cannot study the system in isolation from the rest. To account for global effects, we further test our hypothesis with a more realistic metabolic model that we previously developed (64). This metabolic model comprises reactions collectively annotated as carbon metabolism in *E. coli,* that is, glycolysis, pentose-phosphate pathway, Krebs cycle, glyoxylate shunt, and acetate excretion/import. The model (Fig. 2c) has 34 enzymatic reactions, 26 variable metabolites, and two external metabolites (glucose and acetate). It is encoded as a linear approximation called linlog, which was introduced by (49) as an alternative to classical non-linear rate functions (like Michaelis-Menten; see Materials and Methods for further details). In this formulation, enzyme levels are taken as multipliers of the rates and are stored in the diagonal matrix 𝑬 (equation 1). Thanks to its linearity, the linlog system of equations can be solved analytically at the steady state (equation 5). Consequently, we can use the model to quantify the perturbations in metabolite pools when enzyme abundances change by simply feeding the new enzyme levels into the solution to obtain the steady state metabolite concentrations. Deviations from homeostasis can be summarized by the variation in metabolite concentrations when the division time changes. In brief, we run an optimization of gene positions relative to the ori/ter axis under the evolutionary objective of minimizing the perturbations to metabolite concentrations when the division time varies across a certain number of discrete, biologically meaningful values (from 20 to 120 minutes; see Materials and Methods for details). To this end, we use an optimization tool in R (nlminb) and the following objective function:


(23) $$F=\frac{1}{m}\sum_{i=1}^{m}\frac{\sigma_{i}}{\mu_{i}}.$$

Where $\mu_{i}$ and $\sigma_{i}$ are the mean and standard deviation of the concentration of metabolite i across the tested division times, respectively. F is therefore the average coefficient of variation of metabolite concentrations in the system when τ is changed, and our fitness proxy is proportional to its inverse. We stress here for clarity that this simulation has no mechanistic basis for gene order reorganization and only considers the effect of genome structural variations on metabolic homeostasis by evaluating the objective function; it is therefore not informative on the mechanisms behind this process but can provide information on the result without additional parameters. Clearly, this is only a proxy of a true evolutionary process, since the optimization algorithm can check in advance the gradient of the objective function to perform the best moves at each iteration. In “Simulation of structural rearrangements, we will show that a more realistic model gives a congruent answer but requires much longer simulation times. We note that the algorithm used is a local optimizer, therefore, we may end up with solutions that are not global minima. However, the task in this specific case is to test if under our assumptions gene cluster form, not to find the best possible configuration.

In Fig. 2b, we show the outcome of 100 optimization runs. Several genes consistently form compact clusters at convergence (same shade of gray within a column) as expected by our theoretical considerations. Clusters formed at convergence of different optimizations are not always the same, with some of the genes ending in different or no cluster in different simulations, suggesting the existence of alternative, similarly optimal solutions, and/or an effect from the starting conditions of some genes. Globally, we observe a strong correlation of clusters formed in the optimizations and the pathway of the genes (compare with Fig. 2c). Fig. 2d shows all the iterations for one optimization run, starting at the top with a random gene arrangement that evolves for 250 thousand generations.

One alternative for reducing the perturbations introduced by multiplicity changes induced by replication is the calibration of promoter characteristics; therefore, we implement a second optimization where genes have fixed coordinates, division time can change as in the above experiment, and enzyme levels are determined by a parameter under optimization to mimic the evolution of promoter characteristics. More precisely, enzyme levels in the linlog model are in this case calculated as $E_{i}=n_{i}k_{i}$, where $n_{i}$ is the usual copy number of gene i defined by its static position and the tested division times, and the $k_{i}$s, the parameters under optimization, account for the strength of the promoter. Each iteration starts with random gene orders and ends at convergence as before. Fig. 2e shows that promoters cannot provide the same degree of homeostasis as the optimization of gene positions. We conclude that the formation of gene clusters and operons might indeed represent an optimal evolutionary solution to cope with these metabolic perturbations.

The above considerations would suggest that the selective pressure driving the formation of compact gene clusters and operons is a function of the range of division times attainable by a certain organism; species may therefore experience different levels of selection toward the formation of operons, depending on the ability to change division time over a more or less wide range.

### Simulation of structural rearrangements

**Fig 3:**
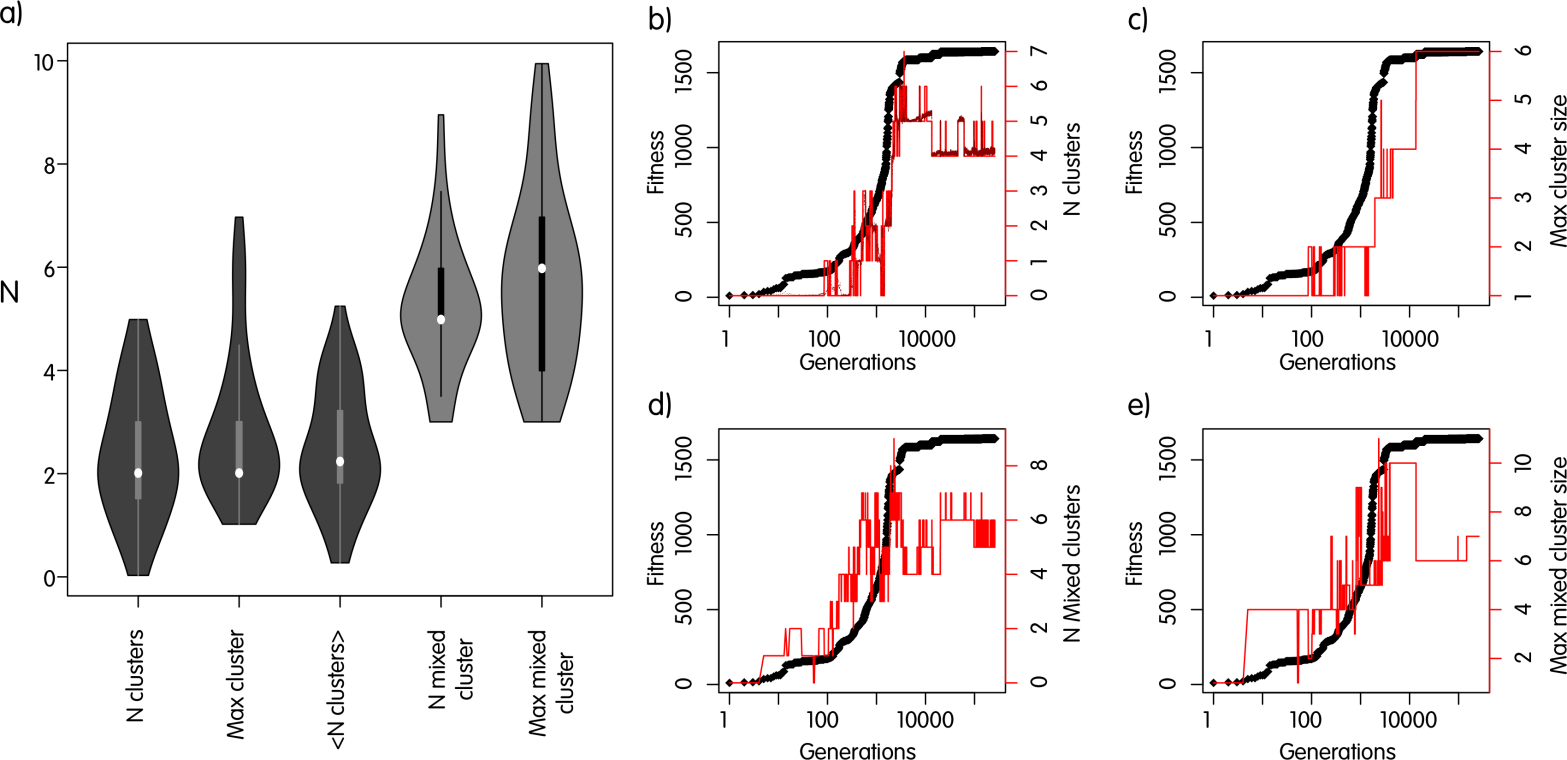
Simulations of gene order evolution with inversions and translocations. (a) Counts and lengths of clusters in 50 independent simulations ran for 100K generations. (**b–**e) Results for one specific simulation after 250K generations. In black, the fitness of the fittest strain in the population. (b) Number of clusters containing only metabolic genes in the fittest strain. A cluster is defined as a contiguous stretch of genes. Dark red dots, mean number of clusters in the population. (c) Number of genes in the largest cluster. (d and e) As panels b and c but for mixed clusters (see definition in Materials and Methods).

The optimization of gene positions discussed above suggests that during evolution, structural rearrangements are able to build gene clusters, under the selective pressure discussed so far. However, the optimization algorithm used above does not explicitly account for molecular mechanism and may therefore provide biased results. One major problem in a realistic scenario is for instance that rearrangements bringing some of the genes at nearby positions could split previously formed clusters, making the optimization of gene positions harder or even impossible. Therefore, we implemented a more realistic scenario where inversions and translocations are considered at the phenomenological level, and we integrated it with our metabolic network model. Previous works studying the formation of clusters from a structural point of view often focus on a single mechanism (inversions or translocations) and on artificial definitions of pathways or other functional interactions of genes. Ballouz et al. (26) have, for instance, demonstrated the ability of inversions to form gene clusters when the fitness is a function of the distance of two target genes belonging to the same virtual pathway. Their fitness function can be interpreted as an abstraction of what we formalized here, but the system is much simpler because the pathway is of two genes only, it is not embedded in a larger metabolic network, and the other genes in the genome are all neutral with respect to rearrangements. Therefore, we simulated a much more realistic scenario, with structural rearrangements taking place in a genome with 600 loci, comprising the 34 genes encoding the enzymes of the metabolic system. In summary, the simulation starts with a population of 10^5^ identical genomes; inversions and translocations take place with a certain constant rate. Once a structural rearrangement takes place in a genome, the fitness is re-calculated by exploiting the metabolic network at different growth rates, and accounting for the multiplicity of genes calculated on the basis of genomic positions with respect to the ori/ter axis, like before. Once all genomes that underwent a structural rearrangement are processed, variants with a fitness less than the 50th percentile are removed from the population, and the surviving genomes are sampled proportionally to their fitness. The new population then undergoes another generation with rearrangements, selection, and so on. It is important to notice that we do not define pathways in advance; therefore, there is not a pre-defined gene order configuration to be achieved. Instead, structural variants with higher fitness will increase in the population, and we trace the formation and number of gene clusters comprising target genes. We also stress that we did not reduce the probability of splitting previously existing clusters as done, for instance, by reference 27. During our simulations, each pair of genes has therefore the same probability of being broken. We highlight that additional simulations suggested that forcing the maintenance of clusters in our system reduced the optimality of solutions, by forbidding better configurations (data not shown). This is clearly impossible to observe when the focus is on a two gene system, but it becomes clear in our case. The inclusion of the aforementioned mechanisms in our simulation framework did not affect the main outcome of our *in silico* evolution experiment. Indeed, as shown in Fig. 3, in all cases tested so far, one or more pure and/or mixed clusters (as defined in Materials and Methods) always formed, thus confirming and extending our hypothesis. It should be noted that the degree of clustering is indeed smaller in the more realistic case, but we demonstrated that the selective force proposed in this work is able to select for gene configurations, also comprising a certain number of clusters, that enable a huge improvement in homeostasis.

### Degree of compaction of functionally related genes correlates to the PTR

One testable consequence of our hypothesis is that species able to achieve larger PTR should experience a more effective selective pressure for clustering functionally related genes. As all species can attain long division times, we can consider the average PTR in laboratory conditions as a proxy of the shortest division time that a species can potentially experience. Two confounding factors in this attempt are horizontal gene transfer and the evolutionary rate of the PTR itself: metabolic operons are easily transferable self-contained modules that are likely advantageous even in species with a very low average PTR; additionally, the PTR calculated from genome sequencing reads is a snapshot of present-day organisms, whereas the degree of gene clustering in genomes reflects a much longer and unknown evolutionary path.

With these drawbacks in mind, we tested our hypothesis by deriving a PS that summarizes the degree of compaction of functionally related metabolic genes in complete genomes (see Material and Methods). The PS of each genome was then compared with the C/τ ratio obtained from genome sequencing data, by using both regression models, t.tests, and phylogenetic correlation in R.

**Fig 4:**
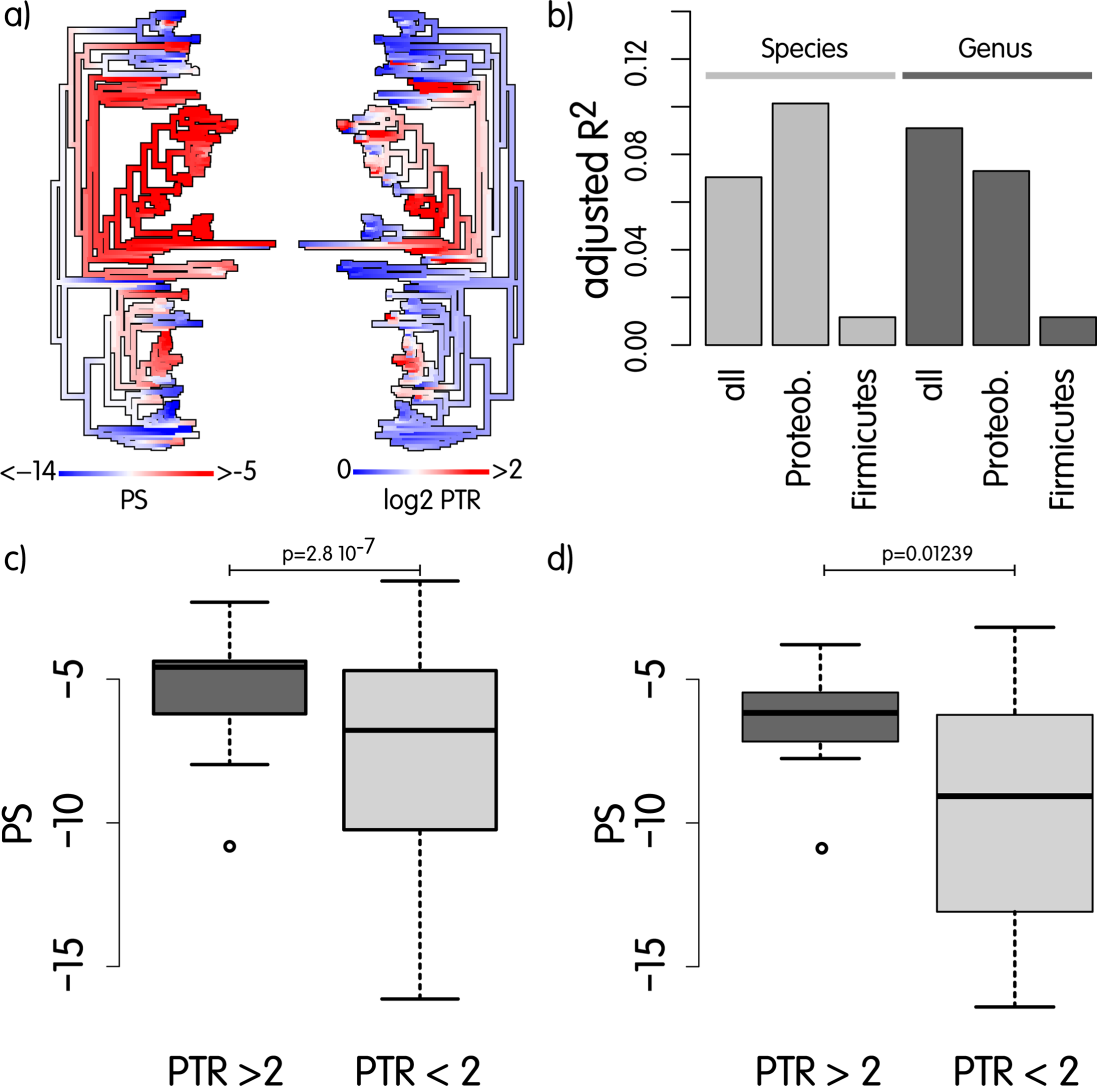
(a) Face-to-face phylogenetic trees with mapped ancestral reconstructions of PS and log_2_PTR. (b) Barplot of the adjusted $R^{2}$ for linear regression models using PS as a predictor of log_2_PTR. All regression coefficients for PS are significant except for firmicutes (data not shown). Species correspond to models exploiting all the organisms in the data set, and genus means that models are built on averaged values across species belonging to the same genus. We also built models for proteobacteria (*N* = 46) and firmicutes (*N* = 29) as they cumulatively represent 86% of the species under analysis; (c) boxplot showing that species having P≤0.01 when testing for PTR≥2 tend to have significantly larger PS. Firmicutes have significantly larger PS (p<2.2e−16) and PTR (p=0.0003766) with respect to the proteobacteria, and this may affect the above pvalue, but (d) which is limited to proteobacteria, shows that the test is still significant without firmicutes.

Figure 4a qualitatively shows the existence of possible covariation of the two traits across the phylogenetic tree in a qualitative way; Fig. 4b confirms the presence of a significant relationship of the PS with C/τ, when using linear regression models; the coefficients are significant when considering all organisms, or the proteobacteria, but not the firmicutes alone. The model explains around 10% of the total variance in the data, which is a consistent fraction if we think about the many additional forces that act on genome organization in much shorter evolutionary times. In Fig. 4c, we strengthen this idea by showing that species with C/τ>1 (p≤0.01) also have a larger PS. Since the firmicutes have PS and C/τ ratios that are significantly above those in proteobacteria, we additionally show in Fig. 4d that the difference is significant even if we limit the test to proteobacteria. The situation of the firmicutes may seem in contrast with our hypothesis. However, when comparing their PS and PTR to the proteobacteria, we found they are both significantly larger $p<2.2\;10^{-16}$ and $p<3.8\;10^{-4}$, respectively. This suggests that the firmicutes likely have a tendency toward high PTR values since their common ancestor, which may explain the thorough optimization of gene organization in this group. Since genomic observations are by definition not independent, we also calculated phylogenetic contrasts on the basis of the tree (Fig. 1) and the data, and then, we calculated their correlation, which is also significant ($R^{2}=0.29$, p=0.00108), indicating that the relationship highlighted above is not caused by phylogenetic correlation.

## DISCUSSION

In this paper, we propose and explore the novel hypothesis that metabolic gene clusters evolve to face metabolite pools perturbations introduced by DNA replication and enable an easier control of metabolic fluxes. Using a set of simulations of increasing complexity that combine MCA with a model of gene multiplicity during replication, we provide the theoretical basis for our hypothesis and motivate the existence of a selective pressure that likely contributed to gene clustering. In doing so, we also disclose a plausible mechanism through which other hypotheses can play a significant role, as they require genes in close proximity. The improvements of this work with respect to previous ones is that since flux and concentration control coefficients are systemic properties, we rely on a realistic metabolic system and integrate it with a genome evolutionary model that explicitly takes into account structural rearrangements. Another important aspect of this work is that we are not using an artificial definition of a metabolic pathway, but, rather, we define a metabolic system potentially containing many overlapping pathways, and we let gene organization evolve on its own based on the effect on metabolite homeostasis. This complicates and slows down considerably the evolutionary process because many structural rearrangements that move some of the genes closer, likely split previously formed clusters. However, this is exactly what happens in real genome evolution where all genes somehow exert an effect on fitness and are therefore not completely free of moving; the genomes where an event breaks a pre-existing cluster will survive if this is compatible with their fitness. Even with these complications, we are able to observe the formation of clusters and a strong increase in the fitness consequent to genome rearrangements. Taken together, our simulations show that introducing the PTR as a controller of enzyme abundances in a metabolic model and fixing an evolutionary meaningful objective such as metabolite homeostasis leads to the formation of gene clusters.

One possible limitation of our hypothesis is the existence of a critical distance separating two genes ($d_{crit}$) such that variants with the genes closer than this threshold do not get any appreciable fitness increase. In brief, and focusing on 2 genes *a* and *b* for clarity, when $d_{a,b}=d_{crit}$ further reductions in distance become practically neutral. In this scenario, additional compaction may be indirectly selected in the population by the negative selection of variants where $d_{a,b}^{\prime }\gg d_{crit}$ if this is associated with a significant decrease in fitness. Moreover, when there are more than two clustered genes, the critical threshold refers to the largest distance, and therefore, further compaction within the cluster could still happen.

**Fig 5:**
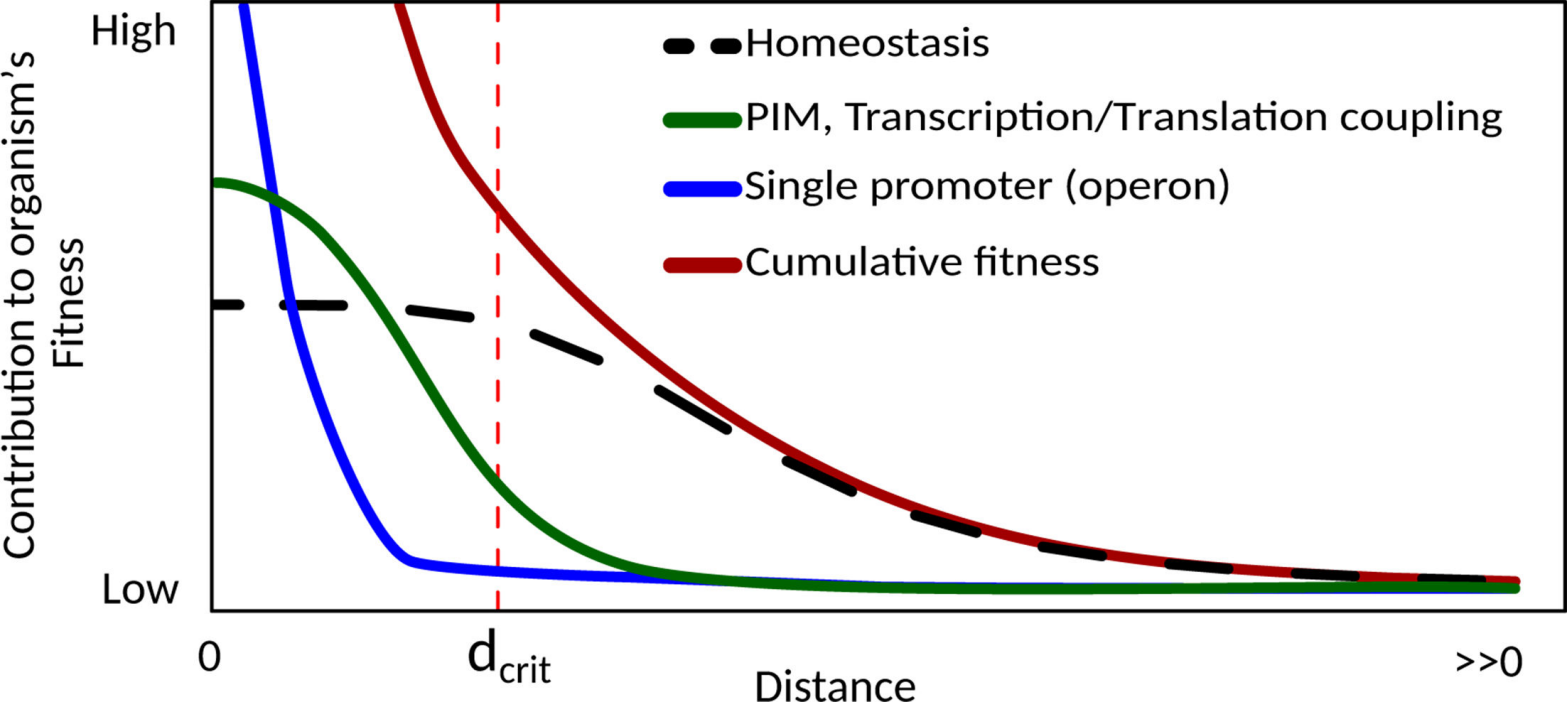
The selective force for better homeostasis is able to explain gene clustering, but once a certain critical threshold $d_{crit}$ for inter-gene distance is reached, further compaction can be driven by other hypotheses as well. Profiles and relative contributions are arbitrary.

In conclusion, we consider our hypothesis instrumental for driving genes at short distances, a prerequisite for many other hypotheses for operon formation. Indeed, once genes get close, other mechanisms can take over. If the genes become a single transcriptional unit, the most important is co-regulation, in addition or otherwise, the production of functionally related proteins in a smaller volume may provide additional advantages. This would make the cluster more resistant in evolutionary time, such that the selection for specific transcription patterns could drive the removal of functionally unrelated genes (Fig. 5). Importantly, we also provide a relative quantification of the weight of our theory on the formation of operons in evolution (roughly 10%), which, to the best of our knowledge, has never been considered when introducing the other theories that deal with operon evolution.

Our theoretical predictions suggest that the larger the average number of replisomes, the stronger the selective pressure toward gene clustering. By deriving a measure of the degree of compaction of metabolic genes for many species, we indeed confirmed that mero-oligoploid species have a significantly and globally more compact organization of metabolic genes. In the future, it would be interesting to experimentally test our theory. For example, by measuring if metabolite pools for pathways that are organized in an operon tend to have a more stable concentration than metabolite pools of pathways with scattered genes. Collecting enough metabolomic experiments at different growth rates for organisms with known genome structures, it should be possible to provide a quantitative interpretation of our gene cluster evolution model.

We believe that our hypothesis has several important repercussions on the way we conceive metabolic operon evolution on the one side and how we see ploidy and DNA replication in bacteria on the other. Indeed, here, we propose that the selective pressure toward gene clustering would be a function of the number of replication forks (summarized here by the PTR) such that mero-oligoploids could be considered major gene cluster formers, whereas others, especially mono-ploids would be subject to reduced pressures in that sense. Nonetheless, fully formed operons still provide significant advantages to the latter, and horizontal gene transfer might be responsible for the diffusion of operons.

Not all cellular processes might benefit from the clustering of their genes into operons, in accordance with the imbalance described in this work; namely, functionally related genes involved in cellular processes other than metabolism might behave differently, yet whenever two proteins work in precise ratios, such as in macro-molecular complexes, a similar effect could be at work, given that the sub-units produced in excess would weigh on the cellular energetic burden, which is in agreement with the results described by reference 27.

## Data Availability

All data and code are available on Mendeley Data, doi: 10.17632/67txzm96jg.1.

## SUPPLEMENTAL MATERIAL

File S1 msystems.00960-24-s0001.pdf Details of the steps related to the integration of the copy numbers in the flux amplification factor.

ASM does not own the copyrights to Supplemental Material that may be linked to, or accessed through, an article. The authors have granted ASM a non-exclusive, world-wide license to publish the Supplemental Material files. Please contact the corresponding author directly for reuse.
