# Supplementary material for: A new selective force driving metabolic gene clustering
Source: mSystems. 2024 Oct 28;9(11):e00960-24. doi: 10.1128/msystems.00960-24 (PMC11629862; doi:10.1128/msystems.00960-24)
Supplement: File S1 — Details of the steps related to the integration of the copy numbers in the flux amplification factor. [file msystems.00960-24-s0001.pdf]

Fondi et al 2024
Supplementary Text

July 12, 2024

1 Flux amplification factor with replication

In the main text, we introduced the following simple model for the abundance of a certain transcript x under the regulation of transcription factor y, and we integrated it with multiplicity of the x locus (n_x), obtaining:

$$\frac{dx}{dt} = n_x f(y, \boldsymbol{p}) - \gamma x, \tag{1}$$

where $\boldsymbol{p}$ indicates the parameters of the regulatory function ($f(y, \boldsymbol{p})$) and γ is a degradation rate. We have seen that the general solution for the steady state abundance of x is:

$$x^{ss} = n_x \frac{f(y, \boldsymbol{p})}{\gamma} = 2^{[(1-p_x)C+D]/\tau} \frac{f(y, \boldsymbol{p})}{\gamma} \tag{2}$$

Which highlights that the level of a transcript at steady state is determined by the abundance of the regulator but also by the division time. Therefore, even if nothing else changes, if the bacterium changes instantaneous growth rate, transcript abundances will be affected accordingly.

Let's now consider the flux amplification factor introduced by [1] to predict the change in pathway flux elicited by a change in enzyme levels:

$$\frac{J^k}{J^0} = \frac{1}{1 - \sum_{i=j}^{m} C_{E_i}^{J^0} \left(1 - \frac{1}{k_i}\right)} \tag{3}$$

where $C_{E_i}^{J_0}$ is the flux control coefficient of enzyme i over the pathway flux in the reference condition (0), and the k_is are relative changes of enzyme abundances: ($k_i = E_i^{\text{new}}/E_i^0$). We can use our solution (Eq. 2) to express k_i for a generic enzyme passing from level x^0 to level x^k as:

$$k_i = \frac{x_i^k}{x_i^0} = \frac{n_i^k f(T^k, \boldsymbol{p})}{\gamma_i^k} \times \frac{\gamma_i^0}{n_i^0 f(T^0, \boldsymbol{p})} = \frac{n_i^k f(y^k, \boldsymbol{p}) \gamma_i^0}{n_i^0 f(y^0, \boldsymbol{p}) \gamma_i^k} \tag{4}$$

We can now introduce this general form for k_i in Eq. 3:

$$\frac{J^k}{J^0} = \frac{1}{1 - \sum_{i=j}^{m} C_{E_i}^{J^0} \left(1 - \frac{n_i^0 f(y^0, \boldsymbol{p}) \gamma_i^k}{n_i^k f(y^k, \boldsymbol{p}) \gamma_i^0}\right)} \,, \tag{5}$$

As expected, the amplification factor depends on both relative changes in (i) copy numbers of the loci, which are functions of division time as indicated by the Helmstetter and Cooper model, (ii) the activity of the regulator, and (iii) the degradation rate.

If all enzymes of the pathway are changed, then $\sum_{i=j}^{m} C_{E_i}^{J^0} = 1$, therefore:

$$\frac{J^k}{J^0} = \frac{1}{\sum_{i=j}^{m} C_{E_i}^{J^0} \frac{n_i^0 f(y^0, \boldsymbol{p}) \gamma_i^k}{n_i^k f(y^k, \boldsymbol{p}) \gamma_i^0}}. \tag{6}$$

If promoter characteristics and degradation rates are the same for all genes, we can move the regulatory part outside the summation and remove the suffix:

$$\frac{J^k}{J^0} = \frac{1}{\frac{f(y^0, \boldsymbol{p}) \gamma^k}{f(y^k, \boldsymbol{p}) \gamma^0} \sum_{i=j}^{m} C_{E_i}^{J^0} \frac{n_i^0}{n_i^k}}, \tag{7}$$

Finally, assuming the shift from condition 0 to k brings no regulatory and degradation changes (therefore the part outside the summation is 1) and that at the same time C and D are unchanged:

$$\frac{J^k}{J^0} = \frac{1}{\sum_{i=j}^{m} C_{E_i}^{J^0} \frac{n_i^0}{n_i^k}} = \frac{1}{\sum_{i=j}^{m} C_{E_i}^{J^0} \; 2^{\frac{\tau^k - \tau^0}{\tau^k \tau^0}[(1-p_i)C+D]}}. \tag{8}$$

Eq. 8 highlights that, if a bacterium faces changes in division time across conditions, then the ensuing variation in copy number can perturb metabolic fluxes. The assumptions used here may appear unreal, but they have the only purpose of isolating the effect due to copy number variations. Without such strong simplifications, replication still represents a confounding factor in the input/output response of classical transcriptional regulation. Moreover, while the form for the metabolite pools amplification factor is more complicated to derive, we note that even if the flux is only marginally affected, there might be larger repercussions on the metabolites. In the main text, we conclude showing that if the position of all genes of the pathway is similar ($p_i \approx p, \forall i$), then a change in division time translates in the following change in pathway flux:

$$\frac{J^k}{J^0} = 2^{\frac{\tau^0 - \tau^k}{\tau^k \tau^0}[(1-p)C+D]} = \frac{n^k}{n^0}, \tag{9}$$

which was the change originally applied to enzyme levels. Since genes are at the same genomic locus, a variation in division time translates in the coordinate change of all enzyme abundances, therefore in a scaling of the fluxes by the same factor, and no perturbation to the metabolite pools.

References

[1] Henrik Kacser and James A Burns. The control of flux. *Symp. Soc. Exp.*, 27:65–104, 1973.
